# *p32* heterozygosity protects against age- and diet-induced obesity by increasing energy expenditure

**DOI:** 10.1038/s41598-017-06209-9

**Published:** 2017-07-18

**Authors:** Yong Liu, Patrick L. Leslie, Aiwen Jin, Koji Itahana, Lee M. Graves, Yanping Zhang

**Affiliations:** 1 Department of Radiation Oncology and Lineberger Comprehensive Cancer Center, University of North Carolina at Chapel Hill, Chapel Hill, NC 27599-7461 USA; 2 Curriculum in Genetics and Molecular Biology, University of North Carolina at Chapel Hill, Chapel Hill, NC 27599-7461 USA; 3 Department of Pharmacology, School of Medicine, University of North Carolina at Chapel Hill, Chapel Hill, NC 27599-7461 USA; 4 Jiangsu Center for the Collaboration and Innovation of Cancer Biotherapy, Laboratory of Biological Cancer Therapy, Cancer Institute, Xuzhou Medical University, Xuzhou, Jiangsu 221002 China; 5 Cancer & Stem Cell Biology Program, Duke-NUS Medical School, Singapore, Singapore

## Abstract

Obesity is increasing in prevalence and has become a global public health problem. The main cause of obesity is a perturbation in energy homeostasis, whereby energy intake exceeds energy expenditure. Although mitochondrial dysfunction has been linked to the deregulation of energy homeostasis, the precise mechanism is poorly understood. Here, we identify mitochondrial p32 (also known as C1QBP) as an important regulator of lipid homeostasis that regulates both aerobic and anaerobic energy metabolism. We show that while whole-body deletion of the *p32* results in an embryonic lethal phenotype, mice heterozygous for *p32* are resistant to age- and high-fat diet-induced ailments, including obesity, hyperglycemia, and hepatosteatosis. Notably, *p32* ^+/−^ mice are apparently healthy, demonstrate an increased lean-to-fat ratio, and show dramatically improved insulin sensitivity despite prolonged high-fat diet feeding. The *p32* ^+/−^ mice show increased oxygen consumption and heat production, indicating that they expend more energy. Our analysis revealed that haploinsufficiency for p32 impairs glucose oxidation, which results in a compensatory increase in fatty acid oxidation and glycolysis. These metabolic alterations increase both aerobic and anaerobic energy expenditure. Collectively, our data show that p32 plays a critical role in energy homeostasis and represents a potential novel target for the development of anti-obesity drugs.

## Introduction

Obesity has gained widespread recognition as a global pandemic caused by the over-nutrition and sedentary lifestyles that pervade most developed countries. From a public health perspective, central obesity is a major risk factor for metabolic disorders, including insulin resistance, hypertension, hyperlipidemia, non-alcoholic fatty liver disease, and cardiovascular disease^1–4^. Although the incidence of obesity is often attributed to unhealthy lifestyle choices, studies have suggested that individual genetic susceptibility determinants play a significant role in predisposition towards obesity in response to over-nutrition^5–7^. Indeed, many genetic determinants contribute to energy homeostasis, which plays a central role in the storage and expenditure of calories consumed. At the cellular level, proper-functioning mitochondria are crucial for energy homeostasis, and deregulation of mitochondrial function has emerged as a major contributing factor to the development of metabolic disorders^8, 9^. Therefore, a better understanding of the molecular basis of the regulation of mitochondria in terms of aberrant fat accumulation is crucial for understanding the pathophysiology of obesity and obesity-related metabolic disorders, as well as for the development of therapeutics to treat these diseases.

p32, also known as complement component 1-q subcomponent binding protein (C1QBP), was originally isolated from the nucleus, where it binds to splicing factor-2 (SF2) and inhibits the binding of SF2 to pre-mRNA^10, 11^. Additional studies revealed that p32 is also expressed on the cell surface, where it binds to complement component 1 (C1q) and mediates the immune response and inflammatory process^12^. Although p32 can be found in multiple cellular compartments, overwhelming evidence suggests that p32 predominantly resides in the mitochondria through its 33-residue N-terminal mitochondria-targeting signal (MTS) sequence^13^. Functional studies have shown that down-regulation of p32 by shRNA results in the suppression of mitochondrial activity and a shift in glucose catabolism from oxidative phosphorylation (OXPHOS) towards glycolysis^14^.

To investigate the *in vivo* function of p32, we generated whole-body p32 knockout mice. Consistent with a previous study^15^, homozygous disruption of *p32* causes early embryonic lethality. However, we unexpectedly discovered that *p32* heterozygous mice, though developmentally normal, display robust resistance to both age- and high-fat diet-induced obesity and maintain insulin sensitivity well into late adulthood. Metabolic analyses revealed that *p32* ^+/−^ mice display higher levels of fatty acid oxidation and aerobic glycolysis, which results in increased energy expenditure and a leaner phenotype. Our study presents compelling evidence suggesting that p32 may be a suitable anti-obesity drug target for the development of inhibitors.

## Results

### Mice haploinsufficient for *p32* are resistant to adulthood obese and diet-induced obesity

**Figure 1:**
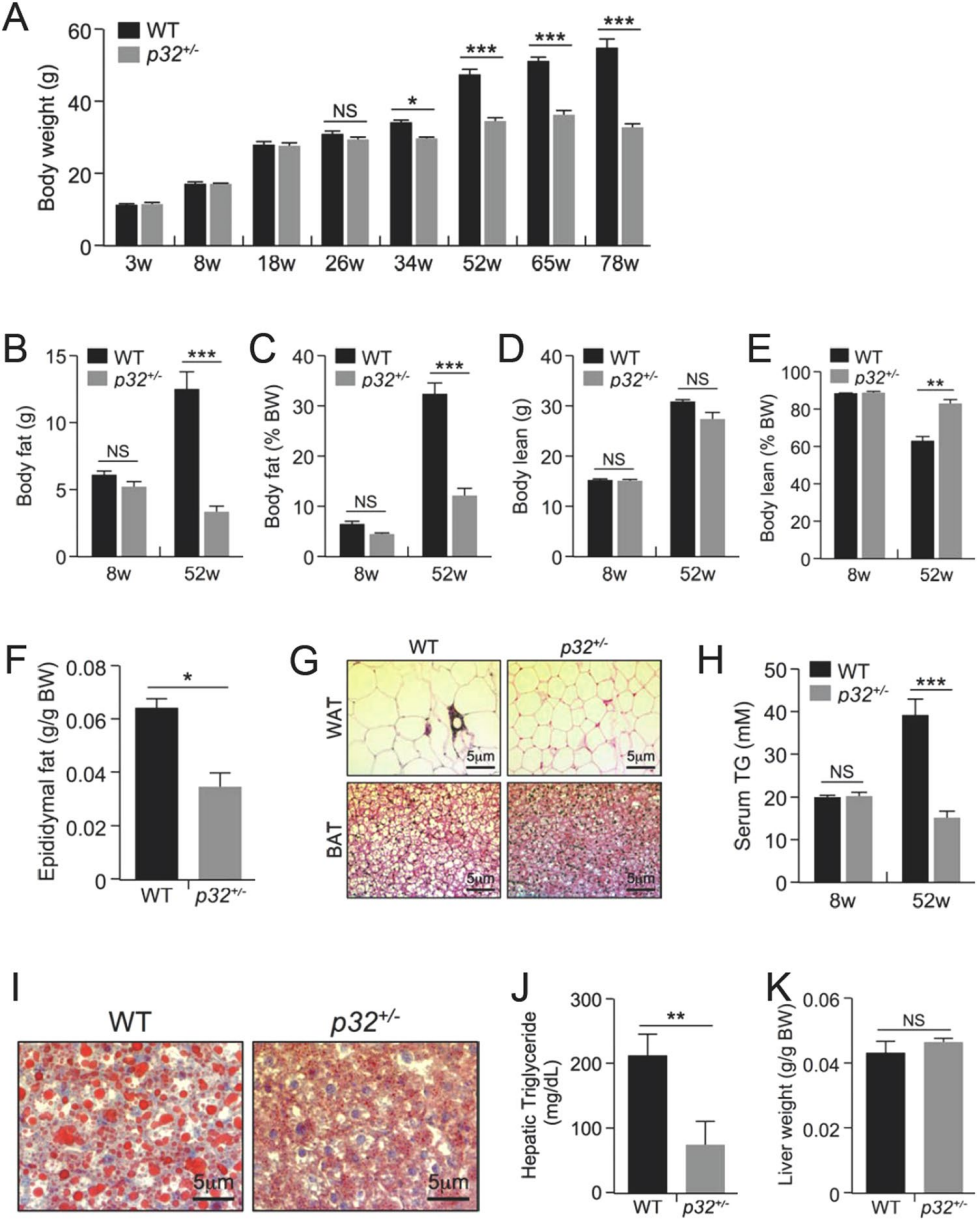
p32 heterozygosity confers resistance to age-associated obesity. (**A**) Body weights of male WT and *p32* ^+/−^ mice fed a normal chow diet (NC) (n = 8). (**B**–**E**) Fat mass (**B**), lean mass (**D**), and the percentages of body weight for fat (**C**) and lean mass (**E**) of 8-week-old and 52-week-old mice fed NC (n = 8). (**F**) Epididymal fat weight of 52-week-old mice fed NC (n = 4). (**G**) Representative hematoxylin and eosin (**H** and **E**) staining of WAT and BAT sections from 52-week-old mice fed NC. (**H**) Serum triglyceride levels of 8-week-old and 52-week-old mice fed NC (n = 4). (**I**) Oil red O staining of liver sections from the 52-week-old mice fed NC. (**J**) Hepatic triglyceride levels of 52-week-old mice fed NC (WT n = 5; *p32* ^+/−^ n = 4). (**K**) Average liver mass of 52-week-old mice fed NC (n = 4).

To investigate the *in vivo* function of p32, we generated *p32* knockout mice by targeted deletion of *p32* exon 3 (Figure S1A). Consistent with a previous report^15^, homozygous deletion of *p32* resulted in early embryonic lethality. However, *p32* heterozygous deletion resulted in mice that are both developmentally normal and fertile, even though p32 protein expression was reduced by half (Figure S1B–D). Interestingly, when maintained on a normal chow diet *ad libitum*, *p32* ^+/−^ mice showed significantly reduced body weight gain starting at approximately 26 weeks of age, whereas the WT littermates continued to gain weight (Fig. 1A). To further study the difference in body weight, we examined body composition of the mice in early (8 weeks) and late (52 weeks) adulthood by magnetic resonance imaging (MRI). At 8 weeks of age, *p32* ^+/−^ mice showed no differences in either body fat (Fig. 1B) or body lean (Fig. 1C) composition relative to WT mice. However, at 52 weeks of age, *p32* ^+/−^ mice displayed marked reduction in both total fat mass (Fig. 1B) and relative fat mass (Fig. 1C). In contrast, the 52-week-old *p32* ^+/−^ mice showed normal amounts of total lean mass compared with their WT littermates (Fig. 1D), which corresponded to a significant increase in relative lean mass after normalization to total body weight (Fig. 1E).

Consistent with the MRI analysis, the epididymal fat pads isolated from 52-week old *p32* ^+/−^ mice weighed 45% less than those of WT littermates (Fig. 1F). Histology analysis revealed smaller adipocytes in both epididymal white adipose tissue (WAT) and interscapular brown adipose tissue (BAT) in the *p32* ^+/−^ mice (Fig. 1G), suggesting that a reduction in size, but not number, of adipocytes is the underlying difference contributing to smaller fat depots and lower body fat content in *p32* ^+/−^ mice.

Because obesity profoundly affects how triglycerides (TG) are metabolized in the body, we compared serum TG levels between WT and *p32* ^+/−^ mice. Consistent with age-related obesity, serum TG levels in 52-week-old WT mice were significantly higher than those of 8-week-old WT mice. However, serum TG levels were essentially no different between younger and older *p32* ^+/−^ mice, and the older *p32* ^+/−^ mice displayed serum TG levels similar to those observed in younger WT mice and significantly lower levels than those of older WT mice (Fig. 1H). To further investigate the differences in adiposity, we assessed fat accumulation in the mouse liver, an organ heavily involved in lipid metabolism. Livers from 52-week-old *p32* ^+/−^ mice exhibited remarkably reduced lipid droplet accumulation compared with those from their WT littermates (Fig. 1I). Consistently, hepatic TG levels in 52-week-old *p32* ^+/−^ mice were approximately 60% lower than those of their WT littermates (Fig. 1J). Of note, although the actual size of the livers of 52-week-old *p32* ^+/−^ mice was smaller than those of the WT mice, the relative size of the *p32* ^+/−^ livers showed no difference from that of the WT mice after normalization to body weight (Fig. 1K).

**Figure 2:**
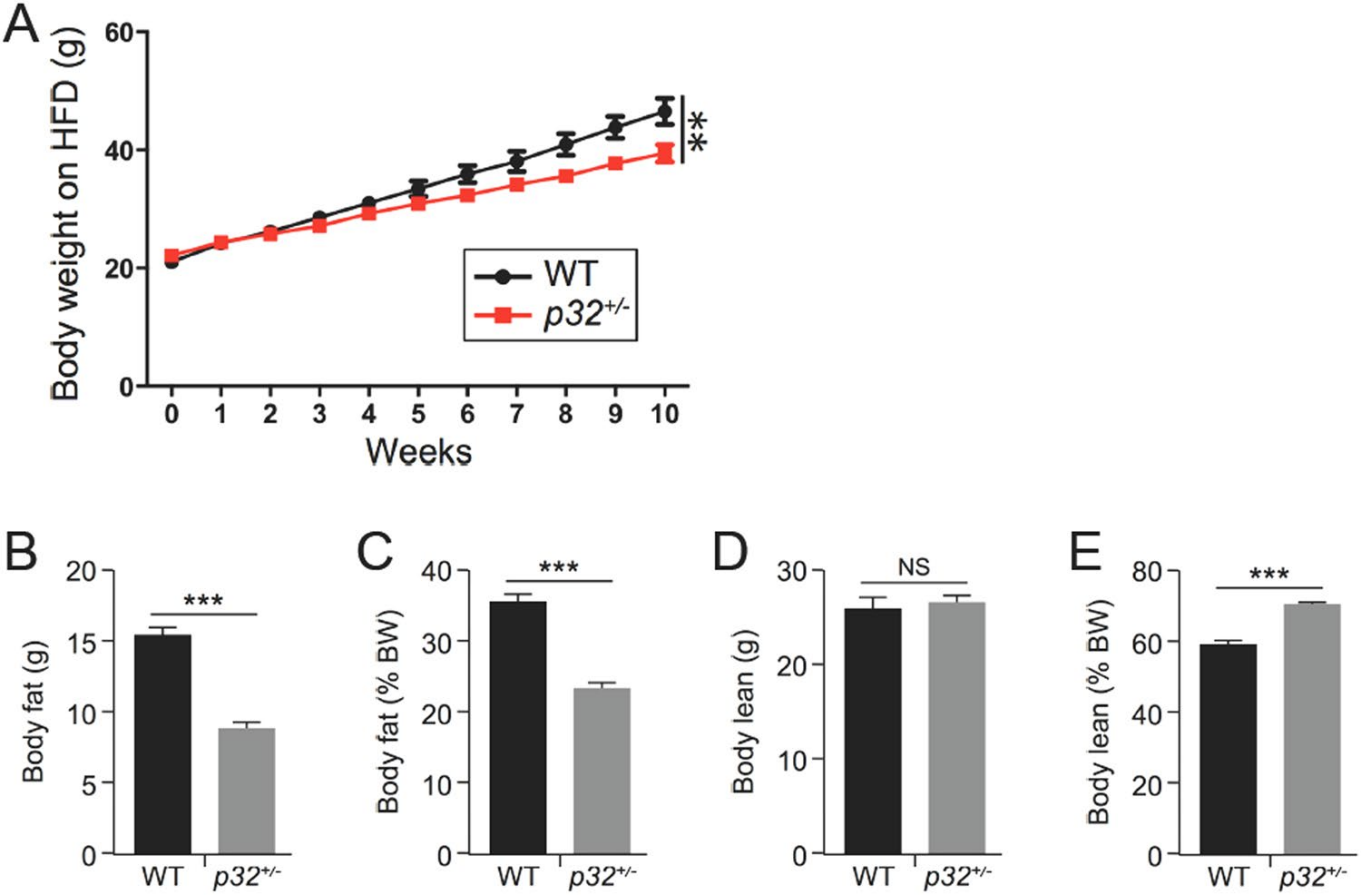
p32 heterozygous mice are resistant to HFD-induced obesity. (**A**) Body weight of 8-week-old WT and *p32* ^+/−^ male mice fed a high-fat diet (HFD) for the indicated amount of time (WT n = 7; *p32* ^+/−^ n = 5). (**B**–**E**) Average total fat (**B**) and lean (**D**) masses and relative fat (**C**) and lean (**E**) masses of WT and *p32* ^+/−^ mice fed an HFD for 10 weeks (WT, n = 7; *p32* ^+/−^, n = 5).

To determine whether the decreased fat accumulation in *p32* ^+/−^ mice can be observed in a diet-induced obesity model, we subjected 8-week-old WT and *p32* ^+/−^ mice to *ad libitum* high-fat diet (HFD) feeding. Similar to the results observed in the development of age-associated obesity, the *p32* ^+/−^ mice gained less body weight (Fig. 2A) and displayed lower levels of both total (Fig. 2B) and relative (Fig. 2C) body fat contents. Moreover, the *p32* ^+/−^ mice showed an unchanged total (Fig. 2D) and an increased relative (Fig. 2E) body lean composition compared with their WT littermates on an HFD. Collectively, these observations demonstrate that heterozygous deletion of *p32* prevents lipid accumulation, which results in resistance to both adulthood obese and diet-induced obesity in mice.

### Mice haploinsufficient for *p32* demonstrate improved glucose tolerance and insulin sensitivity

**Figure 3:**
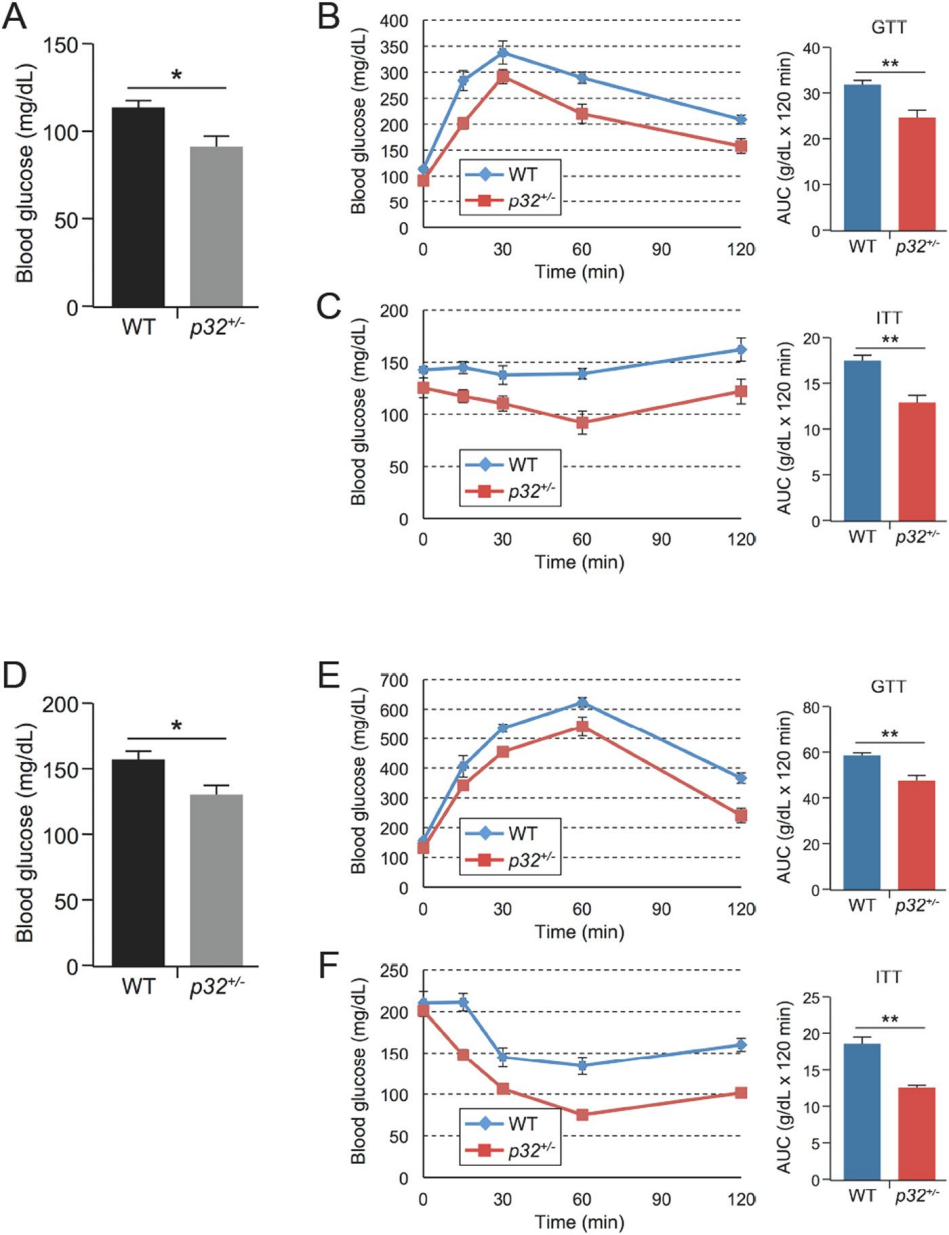
p32 heterozygosity increases glucose tolerance and insulin sensitivity. (**A**) Average fasting blood glucose levels of 52-week-old mice fed NC (n = 6). (**B**) Glucose tolerance test (GTT) and (**C**) insulin tolerance test (ITT) in 52-week-old mice fed NC (n = 6). Bar graphs to the right quantify the areas under the curves (AUC). (**D**) Average fasting blood glucose levels in mice fed an HFD for 10 weeks (WT, n = 7; *p32* ^+/−^, n = 5). GTT (**E**) and ITT (**F**) assays in mice fed an HFD for 10 weeks (n = 5). Bar graphs to the right quantify the areas under the curves (AUC).

Because obesity is associated with the development of insulin resistance, we tested *p32* ^+/−^ mice for their ability to regulate blood glucose levels. Consistent with reduced adiposity, we observed lower fasting blood glucose levels in 52-week-old *p32* ^+/−^ mice compared with their WT littermates (Fig. 3A). To directly assess insulin sensitivity, we subjected mice to a glucose tolerance test (GTT) and insulin tolerance test (ITT). Consistently, we found that the *p32* ^+/−^ mice display improved glucose tolerance (Fig. 3B) and enhanced insulin sensitivity (Fig. 3C) relative to WT mice. Similarly, HFD-fed *p32* ^+/−^ mice also displayed lower fasting blood glucose levels (Fig. 3D), improved glucose tolerance (Fig. 3E), and increased insulin sensitivity (Fig. 3F) relative to the HFD-fed WT mice. Based on these data, we conclude that p32 haploinsufficiency prevents age- and diet-induced obesity, as well as obesity-associated glucose intolerance and insulin resistance.

### Mice haploinsufficient for *p32* show increased caloric intake and energy expenditure

**Figure 4:**
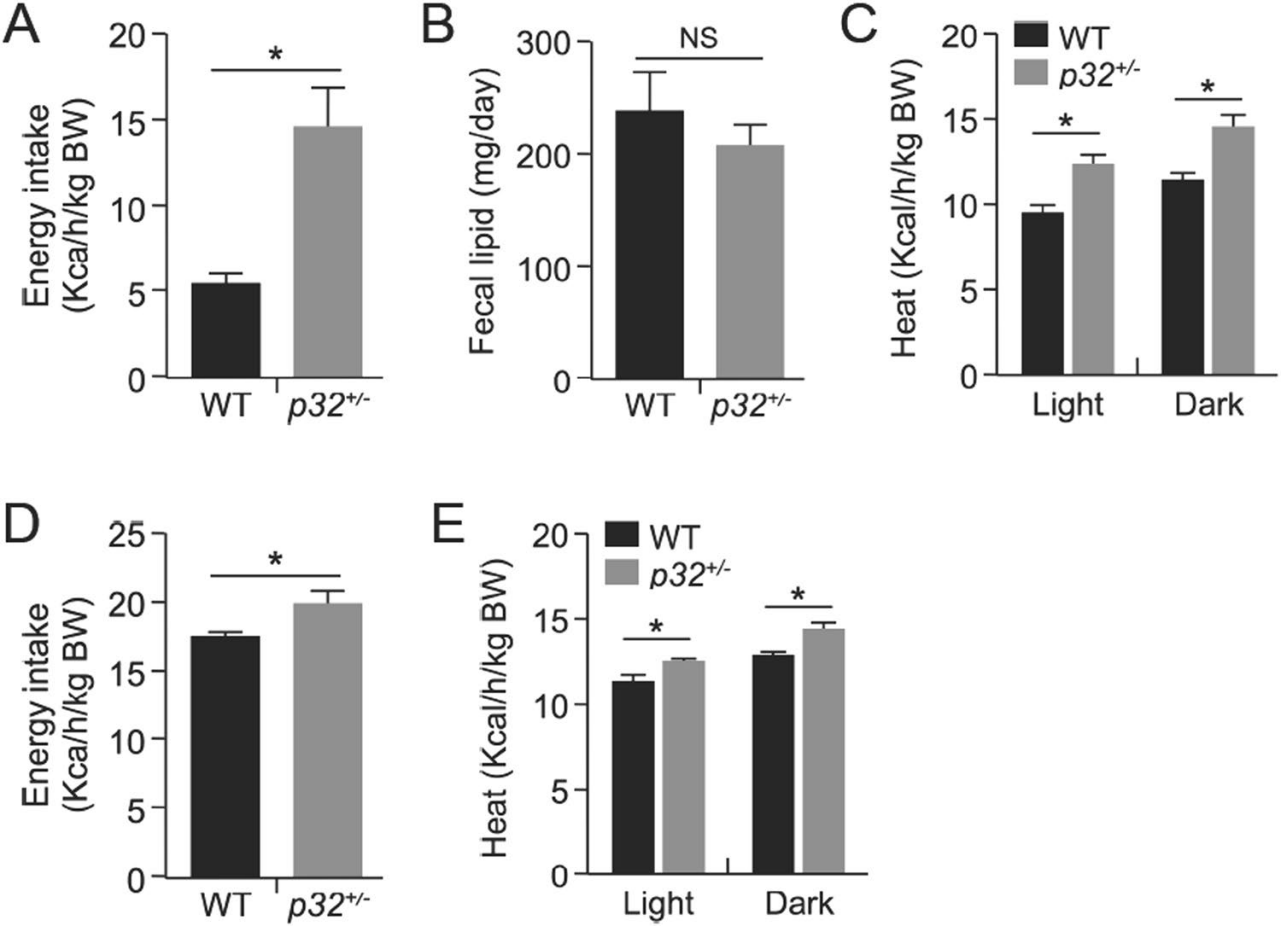
*p32* ^+/−^ mice demonstrate increased caloric intake and energy expenditure. (**A**) Caloric intake in 52-week-old mice fed NC (n = 5). Caloric intake is normalized to body weight. (**B**) Fecal lipid excretion in 52-week-old mice fed NC (n = 5). (**C**) Heat release in 52-week-old mice fed NC (n = 3). (**D**) Caloric intake in mice fed an HFD (WT, n = 7; *p32* ^+/−^, n = 5). Caloric intake is normalized to body weight. (**E**) Heat release in WT and *p32* ^+/−^ mice fed an HFD for ten weeks (n = 3). Statistical comparisons were evaluated by two-way-ANOVA for the indirect calorimetry assay.

Obesity is often a result of increased caloric intake and/or impaired energy expenditure. To understand the underlying mechanism responsible for the resistance of *p32* ^+/−^ mice to fat gain, we examined the energy metabolism in 52-week-old WT and *p32* ^+/−^ mice using a Comprehensive Lab Animal Monitoring System (CLAMS). When fed a normal chow diet *ad libitum*, despite reduced body weight gain, the *p32* ^+/−^ mice demonstrated increased caloric intake (Fig. 4A) compared with WT mice over a 24-h period and showed no difference in fecal lipid excretion (Fig. 4B), suggesting that the *p32* ^+/−^ mice likely experience reduced efficiency in the utilization of energy. To test this possibility, we measured energy expenditure by indirect calorimetry using the CLAMS chamber. Consistent with our hypothesis, *p32* ^+/−^ mice exhibited increased energy expenditure (Fig. 4C) during both light (day) and dark (night) phases compared with their WT controls (adjusted per kg of body weight), indicating that the *p32* ^+/−^ mice display an imbalance in energy uptake and consumption. Consistently, *p32* ^+/−^ mice fed an HFD also showed increased caloric intake (Fig. 4D) and energy expenditure (Fig. 4E). Collectively, these altered metabolic parameters in *p32* ^+/−^ mice indicate that their resistance to obesity occurs primarily as a result of increased energy expenditure due to sub-optimal energy production efficiency.

### *p32* haploinsufficiency results in decreased glucose oxidation and increased fatty acid oxidation

**Figure 5:**
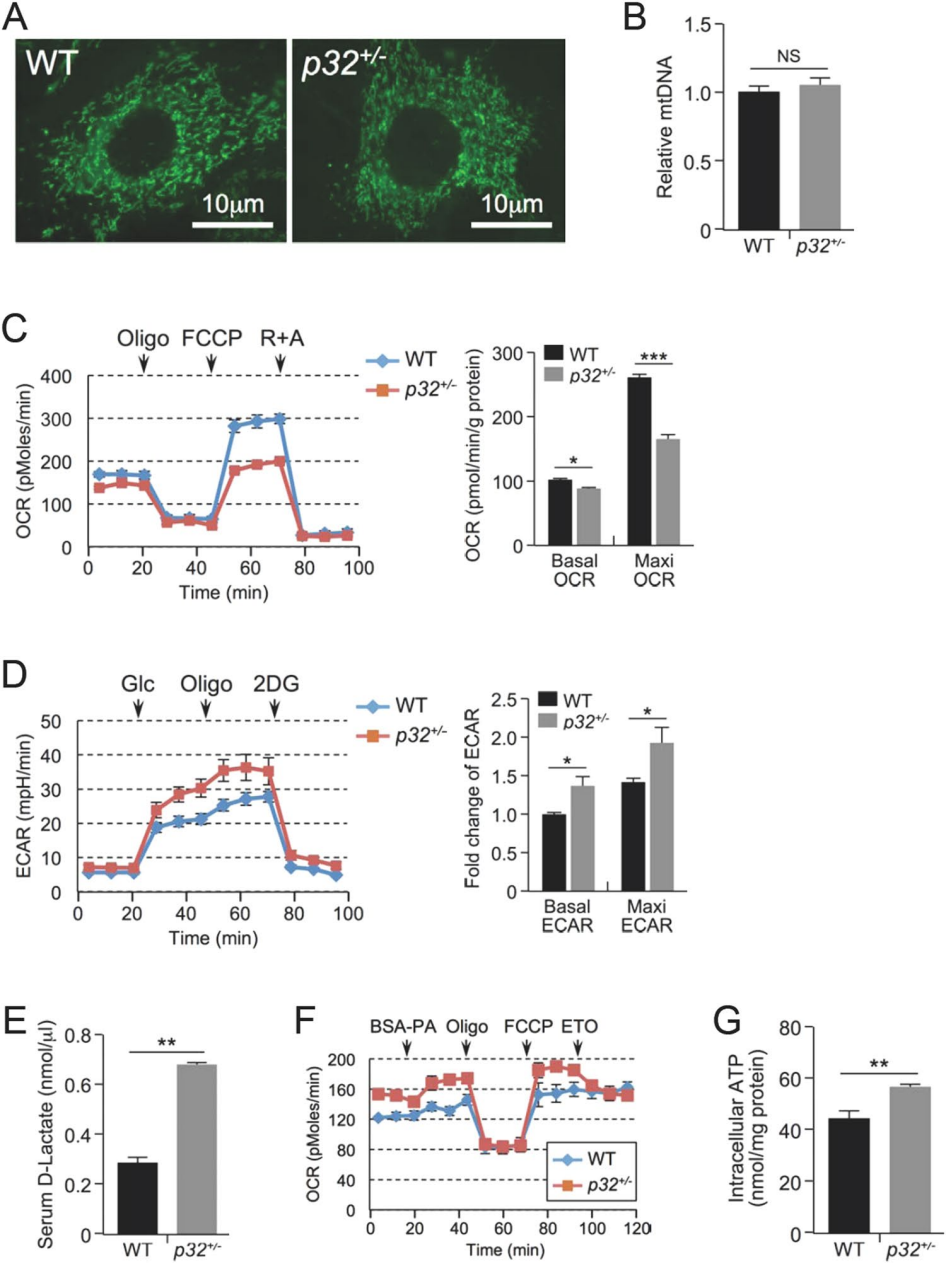
p32 heterozygosity decreases glucose oxidation and increases glycolysis and FAO. (**A**) Representative image of the mitochondria in WT and *p32* ^+/−^ MEFs by Mito-Green staining. (**B**) Relative mtDNA amounts in WT and *p32* ^+/−^ MEFs determined by mtDNA/nuclear DNA ratio using qPCR. mtDNA: mCO1; nDNA: NDUFC1. (**C**) Basal oxygen consumption (basal OCR) and maximum mitochondrial respiration capacity (Maxi OCR, indicated by OCR in the presence of FCCP) in the presence of 25 mM glucose in WT and *p32* ^+/−^ MEFs determined using a Seahorse XF24 analyzer. Left, representative curve; Right, quantification of three independent experiments. (**D**) ECAR in WT and *p32* ^+/−^ MEFs determined using a Seahorse XF24 analyzer. ECAR upon glucose injection (indicated by Glc) minus that of 2DG injection represents the basal ECAR; ECAR with oligomycin injection (indicated by Oligo) minus that with 2DG injection represents maximum glycolytic flux. Left panel, representative curve; Right panel, quantification of three independent experiments. (**E**) Serum lactate levels of 52-week-old mice fed NC (n = 4). (**F**) Basal oxygen consumption (basal OCR) and maximum mitochondrial respiration capacity (Maxi OCR, indicated by OCR in the presence of FCCP) in the presence of 150 μM palmitate in WT and *p32* ^+/−^ MEFs determined using a Seahorse XF24 analyzer. ETO (50 μM) was used as a system control. OCR without ETO minus that with ETO indicated the FAO-specific OCR. (**G**) Intracellular ATP levels in WT and *p32* ^+/−^ MEFs.

Because p32 is a mitochondrial protein^13^ and plays a role in mitochondrial metabolism^14^, we reasoned that *p32* heterozygosity could have an impact on mitochondrial biology. We examined mitochondrial morphology and total mitochondria mass and found no obvious differences in mitochondrial morphology (Fig. 5A) or total amount of mitochondrial mass, as measured by total mitochondrial DNA (Fig. 5B), between WT and *p32* ^+/−^ mouse embryonic fibroblast (MEF) cells. We then determined whether *p32* heterozygosity might affect mitochondrial function in the oxidation of glucose and fatty acids using a Seahorse XF24 analyzer. To measure basal oxidative capacity, oligomycin was injected into the culture wells prior to injection of the uncoupling agent carbonyl cyanide-4-(trifluoromethoxy)-phenylhydrazone (FCCP). When the cells were incubated with medium containing glucose, *p32* ^+/−^ MEFs showed decreased basal and maximal oxygen consumption rates (OCRs) (Fig. 5C). The maximal oxidative capacity, which is indicated by FCCP-sensitive OCR, was decreased by 40%. In compensation for the reduced energy production via oxidative phosphorylation, we found that glucose utilization in *p32* ^+/−^ MEFs was shunted to aerobic glycolysis, which was indicated by an increased extracellular acidification rate (ECAR) (Fig. 5D). To confirm the effect of *p32* heterozygosity on glycolysis, we used 2-deoxyglucose (2-DG), a glucose analog, to inhibit hexokinase, which is the first enzyme in the glycolytic pathway that converts glucose to glucose-6-phosphate. The difference in ECAR between *p32* ^+/−^ and WT MEFs was abolished in the presence of 2-DG (Fig. 5D), supporting the notion that *p32* heterozygosity enhances glycolysis. We also measured the plasma concentrations of lactate in *p32* ^+/−^ mice. Consistent with the observation of higher glycolytic flux in *p32* heterozygous MEFs, serum lactate levels in the *p32* ^+/−^ mice were increased by more than two-fold compared with WT mice (Fig. 5E).

Next, we tested mitochondrial activity with respect to the oxidation of fatty acids, the main alternative fuel source in animals under normal feeding conditions. Interestingly, despite reduced glucose oxidation, *p32* ^+/−^ MEFs showed increased OCR when cultured in medium containing BSA-conjugated palmitic acid (Fig. 5F), suggesting that they are predisposed to higher levels of fatty acid oxidation (FAO) for the production of ATP. Indeed, *p32* heterozygosity resulted in increased ATP production relative to WT cells (Fig. 5G). These data suggest that *p32* haploinsufficiency causes an imbalance in mitochondrial metabolism, which results in a reduction in glucose oxidation that is compensated for by increased FAO.

## Discussion

**Figure 6:**
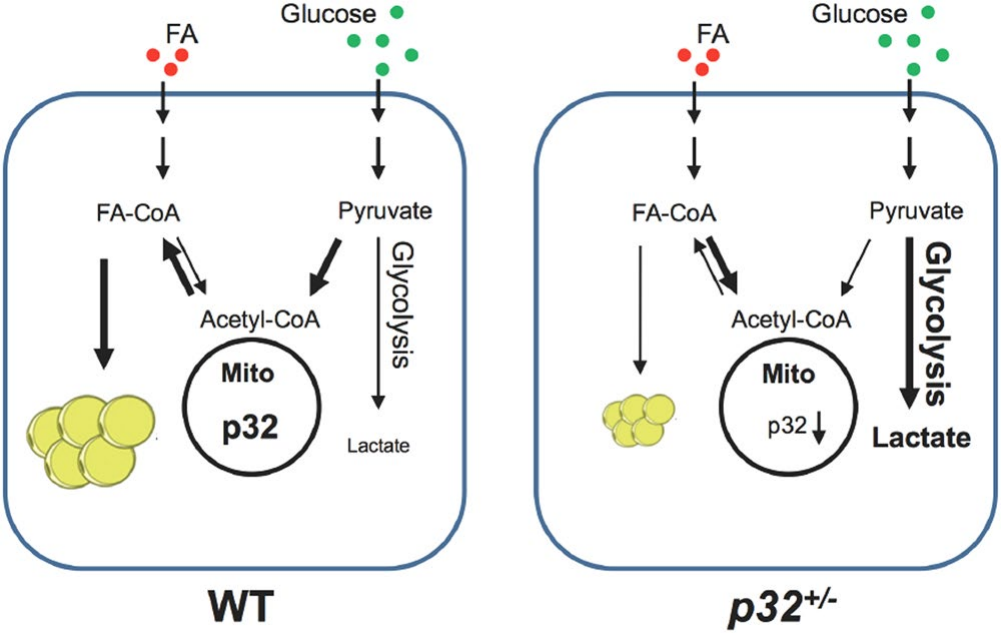
p32 heterozygosity confers resistance to obesity by remodeling glucose and lipid metabolism.

To elucidate the physiological functions of p32, we generated and studied *p32* knockout mice. Interestingly, heterozygous *p32* deletion in mice results in remarkable resistance to age-related and diet-induced illnesses such as obesity, hepatosteatosis, and insulin resistance. Obesity in mice, both age-related and diet-induced, occurs when acetyl-CoA production exceeds acetyl-CoA consumption through mitochondrial OXPHOS, leading to elevated lipogenesis and increased body fat deposition. The *p32* ^+/−^ mice showed reduced glucose oxidation, possibly because of the suppression of the function of mitochondrial complex I^14, 15^, and compensatory increases in glycolysis and FAO to make up for reduced ATP production due to the attenuation of OXPHOS. We postulate that reduced p32 levels results in alterations in mitochondrial metabolism and enhances both aerobic and anaerobic energy expenditure, which increases energy consumption and ameliorates adulthood obese and diet-induced obesity in the *p32* ^+/−^ mice (Fig. 6).

Mitochondria are central organelles for energy metabolism, as they link the Krebs cycle, which occurs in the mitochondrial matrix, to the electron transport chain (ETC), which is present in the inner mitochondrial membrane. In our study, *p32* heterozygosity resulted in increased FAO and decreased glucose oxidation, two processes that rely on the ETC-OXPHOS machinery for energy production. Moreover, down-regulation of *p32* by shRNA or disruption of the *p32* gene results in the down-regulation of mitochondrial complex I, while complex II remains active and at normal levels^14, 15^. Thus, inactivation of complex I activity by p32 ablation largely blocks electron transfer from NADH to the downstream ETC components, which prevents the complete oxidation of glucose (Fig. 5C). Moreover, because glucose-derived succinate also becomes depleted in the absence of glucose oxidation, FADH_2_ derived from FAO becomes the only available electron donor for ETC-OXPHOS production when the *p32* gene is disrupted. Thus, it is plausible that FAO is up-regulated to generate FADH_2_, which delivers electrons directly to complex II and thereby bypasses complex I, to meet cellular demands with respect to ATP production, which consequently results in increased aerobic energy expenditure in *p32* ^+/−^ mice (Fig. 4). Similar metabolic signatures and obesity-resistant phenotypes have also been observed in mouse models in which other critical mitochondrial function-related genes, such as AIF^16^, TFAM^17^ and Cox6a2^18^, have been disrupted. These observations suggest that the mitochondria, as the energy plant of the cell, are important to maintain the exquisite balance between glucose and lipid metabolism and thus to control lipid storage.

It is intriguing that the *p32* ^+/−^ mice show a significant increase in food consumption but only a 20% increase in the oxygen consumption (VO_2_), suggesting that energy expenditure may be underestimated in the leaner *p32* ^+/−^ mice. Oxygen consumption estimates metabolic heat production and is commonly used to represent energy expenditure. However, oxygen consumption measurements are not always proportional to energy expenditure. For example, when the contribution of anaerobic glycolysis to ATP production is large^19^, such as in patients with cancer-related cachexia^20^ or athletes performing intense exercise^21^, pyruvate is rapidly converted to lactate, anaerobic heat is released, and the VO_2_ no longer reflects total energy expenditure levels. In the case of *p32* ^+/−^ mice, glucose is predominantly metabolized through glycolysis, and a large amount of lactate is produced in *p32* ^+/−^ mice (Fig. 5D,E), suggesting that the actual energy expenditure is much higher when considering the combined energy expenditure contributions from both oxygen consumption and anaerobic heat release. Likewise, under conditions of metabolic acidosis (Fig. 5D,E), the respiratory exchange ratio (RER), calculated by VCO_2_/VO_2_, is an overestimation and is no longer appropriate to indicate the fuel source of catabolism because the organism tends to acquire ATP through glycolysis, which inflates the RER in a biased manner. Indeed, we observed a significantly higher RER in *p32* ^+/−^ mice compared with their wild-type littermates (Figure S2). Furthermore, accumulated lactate is reconverted to glucose and pyruvate by gluconeogenesis-competent cells in the liver, which requires further consumption of ATP and likely constitutes a futile cycle. This additional use of ATP to recycle lactate likely contributes to the inefficient utilization of energy in *p32* ^+/−^ mice. Taken together, the underestimated energy expenditure and futile metabolic cycles additively contribute to the leaner phenotype in *p32* ^+/−^ mice.

Most interestingly, the leaner but otherwise normal phenotype of *p32* ^+/−^ mice offers a potential drug target that can be exploited therapeutically in anti-obesity efforts. The striking difference in fat accumulation in *p32* ^+/−^ mice with no apparent health deficiencies illustrates the potential of p32 as an anti-obesity target. Moreover, a recent drug screen yielded several compounds that bind to p32 with low-micromolar affinity^22^. Taking into account the reduced fat accumulation with the loss of only one copy of *p32*, even very low-affinity p32 inhibitors could be effective for the prevention of fat accumulation over the long term. Additionally, because the crystal structure of p32 has been reported^23^, rational drug design and optimization are realistic options. Future studies analyzing the effect of various p32 inhibitors on the health and fat accumulation in animal models could offer valuable data and promising drug candidates for anti-obesity clinical trials.

In conclusion, while a multitude of *in vitro* studies have identified potential functions of p32, using a *p32* heterozygous knockout mouse model, we offer the first physiological insight into the function of p32 in the regulation of energy homeostasis. We show that the deletion of a single allele of *p32* is sufficient to produce resistance to obesity and prolong the sensitivity of mice to insulin. This newly discovered obesity-resistant phenotype conveyed by *p32* heterozygosity offers an intriguing therapeutic target in the fight against obesity that warrants further study.

## Methods

### Mice and cell culture

To generate p32^+/−^ mice, a 7.5-kb mouse p32 gene-containing fragment was subcloned into the pBluescript II SK vector and was used to create a p32 loxP targeting vector. This vector was constructed by inserting an FRT-PGK-Neo-FRT-loxP cassette into intron 2 and another loxP site into intron 3, followed by a PGK-Diphtheria toxin A (DTA) expression cassette at the 3′ end of the construct to be used as a negative selection marker (Figure S1A). WT and p32^+/−^ mice on C57BL/6 backgrounds were bred and maintained in standard cages on a 12:12 h light/dark cycle with free access to normal chow (NC) or a high-fat diet (HFD, Research Diets) containing 60% fat. All mice were handled in strict accordance with our animal protocol (12–328), which was approved by the Institutional Animal Care and Use Committee (IACUC) at the University of North Carolina Animal Care Facility. Mice used for HFD experiments were fed with NC until eight weeks of age, after which they were fed with an HFD for another ten weeks. Mouse embryonic fibroblasts (MEFs) were isolated from embryos at embryonic day (E) 13.5 and were cultured in DMEM medium supplemented with 10% FBS, penicillin (100 IU/ml), and streptomycin (100 µg/ml) in a 37 °C incubator with 5% CO_2_ and 3% O_2_.

### Metabolic analyses

Body weights were recorded weekly. Body composition was assessed using an EchoMRI-100 quantitative magnetic resonance whole body composition analyzer (Echo Medical Systems). To determine energy intake, mice were housed individually for one week to allow for environmental habituation, after which food consumption was determined by weight every 24 hours for seven consecutive days. Energy intake in calories was calculated by multiplying the number of grams of normal chow diet or HFD consumed daily by 3.93 kcal/gram or 5.49 kcal/gram, respectively. For metabolic studies, mice were placed individually in metabolic chambers of an Oxymax system (Columbus Instruments) and were allowed to acclimate during a 24-hour period. Then, readings were taken for five days. Water and food consumption data as well as energy expenditure were obtained and were normalized to body weight. For glucose tolerance tests, 16-hour-fasted mice were injected intraperitoneally with D-glucose (2.5 g/kg body weight). For insulin tolerance tests, 6-hour-fasted mice were injected with insulin (0.75 U/kg). Blood glucose concentrations were determined from tail vein blood samples collected at the designated time points and were measured using an ACCU-CHEK Aviva glucometer (Roche, Basel, Switzerland). Serum TG (Stanbio, Boerne, TX) and lactate (Biovision Inc., Milpitas, CA) levels were measured using colorimetric kits based on the protocol recommended by the manufacturer. For the hepatic TG measurement, we used a modified protocol that was previously described by Ferraz^24^. Briefly, snap-frozen liver samples (30–50 mg) were homogenized using a motorized chuck and Teflon pestle in 1% Triton-x-100 in PBS at a concentration of 1 mg/dL. Samples were centrifuged for 1 min at 1500 × g, and the supernatant, including the fatty layer, was transferred to a fresh microcentrifuge tube. Supernatants were assayed for triglyceride content using the StanBio Triglyceride Liquicolor kit using the manufacturer’s recommended protocol (cat. no. 2100–430).

### Oxygen consumption and glycolytic analysis

The oxygen consumption rate (OCR) and the extracellular acidification rate (ECAR) were evaluated using a Seahorse XF24 Analyzer (Seahorse Bioscience) as described previously^25^. Briefly, 6 × 10^4^ MEF cells were seeded in each well of an XF24 cell plate 24 h before the assay. The basal levels of OCR (for the purpose of glucose oxidation) and ECAR were measured in XF assay medium containing 4.5 g/L glucose followed by the addition of oligomycin (1 μM), which allows for the assessment of the maximal glycolytic capacity (ECAR), or by the sequential addition of oligomycin (1 μM), FCCP (0.5 μM) (maximal OXPHOS), and antimycin A (2 μM) plus rotenone (1 µM) to measure the OCR. For FAO measurement, 6 × 10^4^ MEF cells were seeded 24 h before the assay. FAO was measured by sequential injection of BSA-conjugated palmitate (150 μM), oligomycin (1 μM), FCCP (0.5 μM), and etomoxir (ETO, an irreversible inhibitor of carnitine palmitoyltransferase-1, 50 μM) in KHB (Krebs-Henseleit) buffer.

### Histology

Epididymal fat pad and interscapular brown adipose tissue samples were fixed and paraffin-embedded. Tissue sections were subjected to standard hematoxylin and eosin staining. For oil red O staining, mouse liver tissues were fixed in 10% neutral formalin for 24 h and then were submerged in 20% sucrose for 2 days. The liver pieces were then frozen in optimal cutting temperature (OCT) solution, and 5-µm-thick tissue sections were cut. Oil red O staining was conducted according to the standard protocol used in the Histology Research Core Facility at the University of North Carolina at Chapel Hill.

### Western blotting

Procedures and conditions for immunoblotting were previously described^26^. Briefly, mouse tissues were homogenized and lysed in tissue lysis buffer (50 mM Tris-HCl, pH 7.4, 150 mM NaCl, 2 mM EDTA, 2 mM EGTA, 0.2% Triton X-100, 0.3% NP-40, and protease inhibitor cocktail). Goat polyclonal anti-p32 (sc-10258, Santa Cruz, Dallas, TX) antibody was purchased commercially.

### Statistical analysis

Data were analyzed using Graph Pad Prism 5.0 software (La Jolla, CA). Statistical comparisons were evaluated using an unpaired Student’s *t-*test if not otherwise specified. P-values < 0.05 were considered statistically significant (*, **, and *** are used to indicate statistical significance corresponding to p-values < 0.05, 0.01, and 0.001, respectively). All results are presented as the means ± standard deviation.

## Electronic supplementary material

Supplemental Information
