# Supplementary material for: p32 heterozygosity protects against age- and diet-induced obesity by increasing energy expenditure
Source: Sci Rep. 2017 Jul 18;7:5754. doi: 10.1038/s41598-017-06209-9 (PMC5516014; doi:10.1038/s41598-017-06209-9)
Supplement: Supplementary file 1 — Supplemental Information [file 41598_2017_6209_MOESM1_ESM.pdf]

p32 heterozygosity protects against age- and diet-induced obesity by increasing energy expenditure

Yong Liu[1,4], Patrick L. Leslie[1,2], Aiwen Jin[1], Koji Itahana[1,§], Lee M. Graves[3], and Yanping Zhang[1,4,¶]

[1]Department of Radiation Oncology and Lineberger Comprehensive Cancer Center

[2]Curriculum in Genetics and Molecular Biology

[3]Department of Pharmacology, School of Medicine, University of North Carolina at Chapel Hill, Chapel Hill, NC 27599-7461, USA

[4]Jiangsu Center for the Collaboration and Innovation of Cancer Biotherapy, Laboratory of Biological Cancer Therapy, Cancer Institute, Xuzhou Medical University, Xuzhou, Jiangsu 221002, China

[§]Current address: Cancer & Stem Cell Biology Program; Duke-NUS Medical School; Singapore

[¶]To whom correspondence should be addressed: E-mail: ypzhang@med.unc.edu; Tel: 919-966-7713

Running Title: p32 heterozygosity prevents obesity

Figure S1

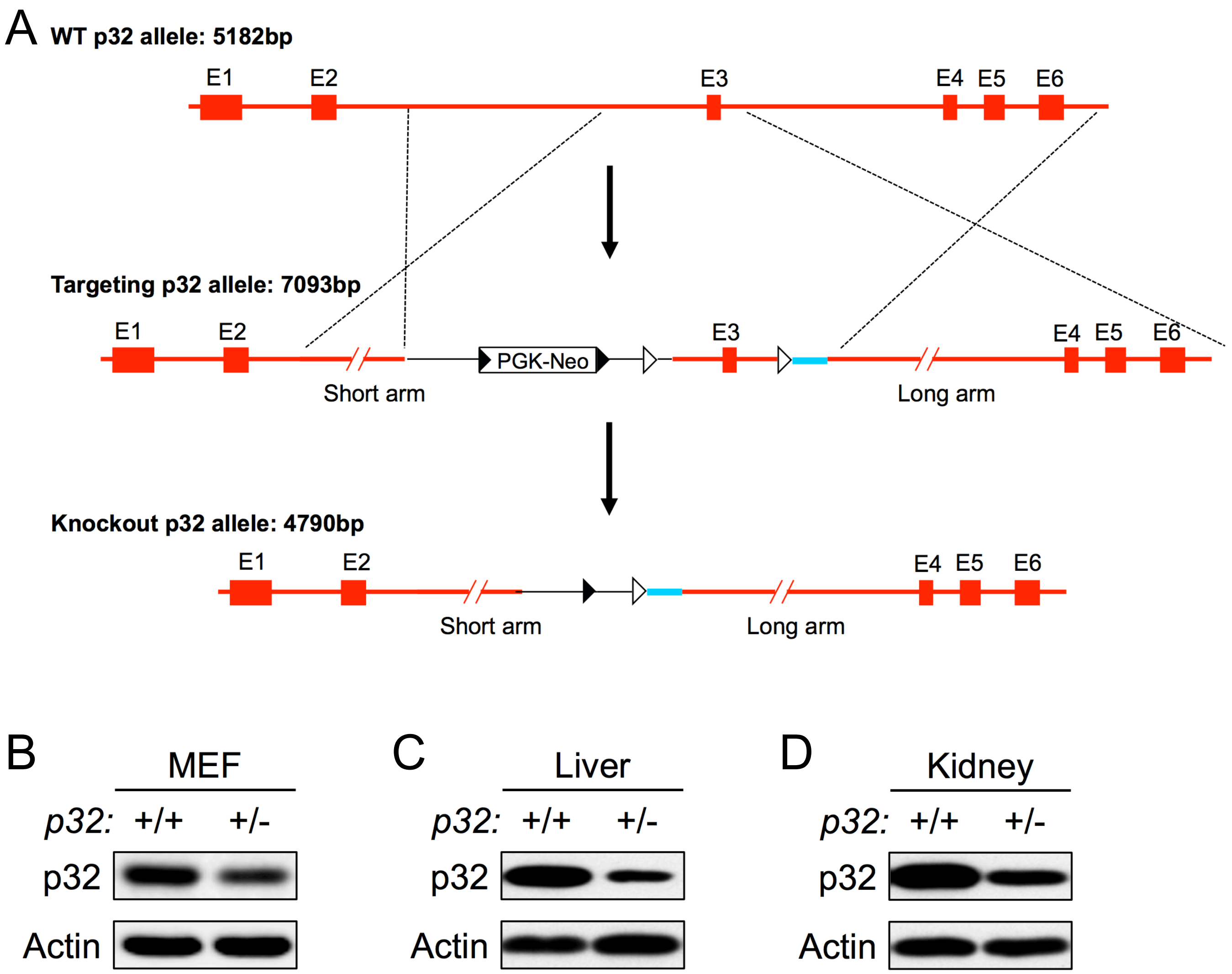
A
WT p32 allele: 5182bp
E1
E2
E3
E4
E5
E6
Targeting p32 allele: 7093bp
E1
E2
PGK-Neo
E3
E4
E5
E6
Short arm
Long arm
Knockout p32 allele: 4790bp
E1
E2
E4
E5
E6
Short arm
Long arm
B
MEF
p32: +/+ +/-
p32
Actin
C
Liver
p32: +/+ +/-
p32
Actin
D
Kidney
p32: +/+ +/-
p32
Actin

Figure S2

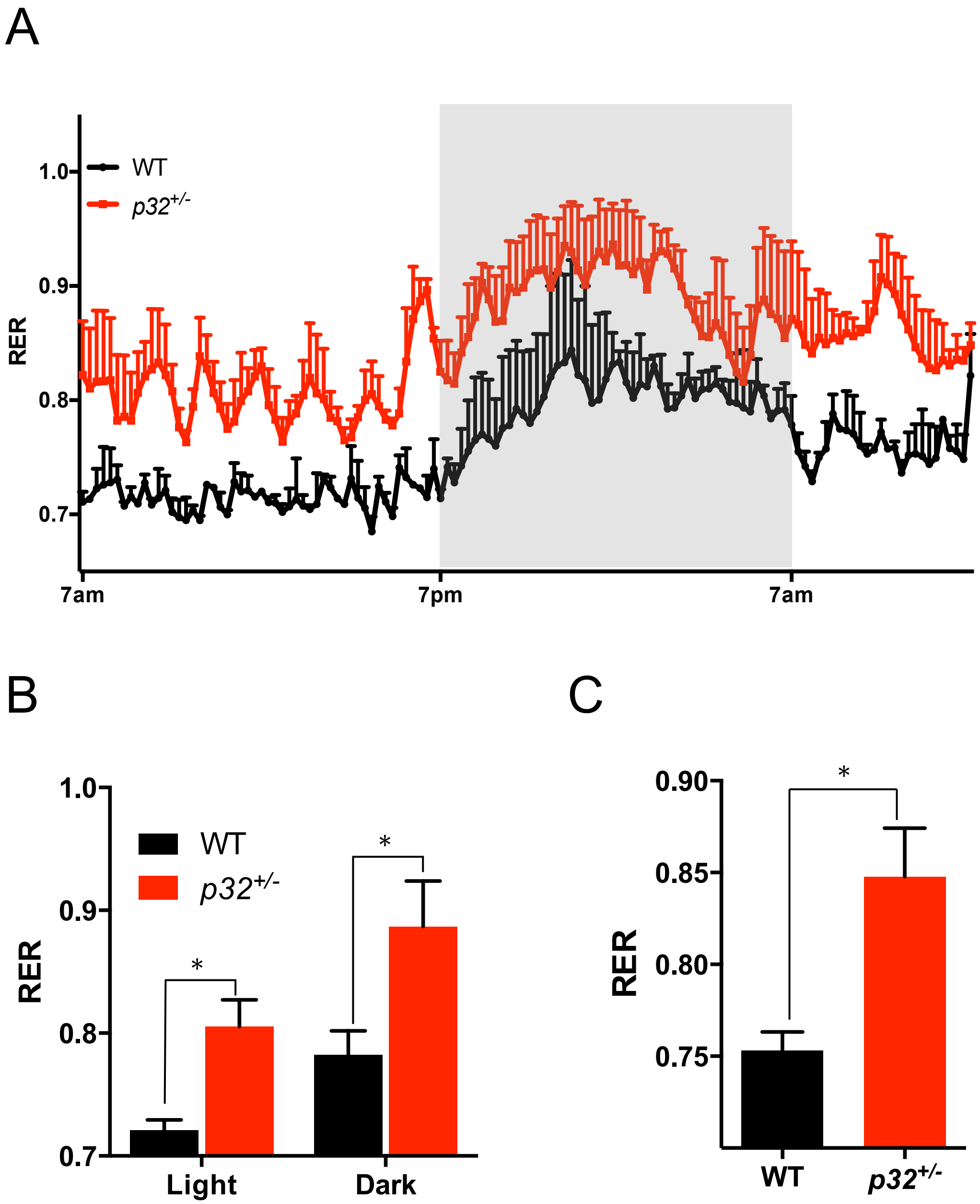
A
WT
p32+/-
RER
1.0
0.9
0.8
0.7
7am
7pm
7am
B
1.0
0.9
0.8
0.7
RER
WT
p32+/-
*
*
Light
Dark
C
0.90
0.85
0.80
0.75
RER
*
WT
p32+/-

Supplemental Figure Legends

Figure S1. Generation of p32 knockout mice.

(A) Schematic representation of the targeting vector for deletion of exon 3 in the mouse *p32* gene.

(B-D) p32 protein expression in MEF cells (B), and liver (C) and kidney (D) tissues.

Figure S2. RER of 52-week-old WT and *p32*$^{+/-}$ mice.

WT and *p32*$^{+/-}$ mice were fed with a normal chow diet for 52 weeks. (A) Respiratory Exchange Ratio (RER) is calculated by CO_2 production over oxygen uptake (CO_2/O_2), which was measured with an indirect calorimetry chamber over the indicated time period. The statistical comparisons were evaluated using a two-way ANOVA (n=3, p=0.028).

(B) Average RER in light (7am through 7pm) and dark cycles (7pm through 7am), n=3.

(C) Average RER over a 24-hour period, n=3.
